# Surrogate-Based EM Design of RF and Microwave Components: A Systematic Review of Workflow Roles, Inverse Design, Multifidelity, and Active Learning

**DOI:** 10.3390/s26082504

**Published:** 2026-04-18

**Authors:** Maria Prousali, Stelios Tsitsos

**Affiliations:** Department of Computer, Information, and Telecommunications Engineering, International Hellenic University, GR-62124 Serres, Greece or mariprou@hotmail.gr

**Keywords:** systematic literature review, surrogate modeling, RF and microwave design, computational electromagnetics, inverse design, multifidelity modeling, active learning, design optimization, surrogate-assisted optimization

## Abstract

**Highlights:**

**What are the main findings?**

- Optimization-based inverse design is the most commonly used approach, whereas multifidelity and active learning are less frequently adopted.

**What is the implication of the main finding?**

- A unified workflow integrating surrogate modeling, inverse design, multifidelity interaction, and active learning has not yet been demonstrated in the RF and microwave literature.

**Abstract:**

Surrogate models have been increasingly used to reduce the computational cost of electromagnetic (EM) design in RF and microwave components. However, component types, surrogate model families, and design workflows vary substantially across the literature. This systematic review provides a structured synthesis of surrogate-assisted EM design and optimization for RF and microwave applications. A Scopus-based screening process was employed to identify 180 journal articles published between 2012 and February 2026. After eligibility assessment, 126 studies were included in the final review corpus, whereas 54 were excluded. Six previous review articles were used separately for contextual positioning. The studies included were classified according to component category, surrogate model family, surrogate usage mode, inverse-design approach, multifidelity integration, active-learning adoption, and workflow function. The results showed that antennas and filters dominate the literature, whereas the Gaussian process or Kriging models and neural networks are the most frequent surrogate families. Optimization-based inverse design is the most commonly used, whereas multifidelity and active learning are less common. Overall, the included literature indicates that surrogate-assisted design is widely represented in RF and microwave design studies. However, no study in the included literature corpus has implemented a unified workflow that combines surrogate modeling, inverse design, multifidelity interaction, and active learning.

## 1. Introduction

Surrogate-assisted modeling and optimization have become increasingly important in RF and microwave engineering because they reduce the computational load associated with full-wave electromagnetic (EM) analyses. Different component classes, modeling strategies, and automation levels have been reported in the literature. Therefore, a structured synthesis is essential to clarify the use of surrogate models in EM-driven design workflows.

### 1.1. Background and Motivation

Previous reviews have examined machine learning (ML) and surrogate-related methods in electromagnetics from several different perspectives. Chen et al. [1] reviewed deep learning (DL) approaches for inverse-scattering problems, emphasizing state-of-the-art methods and combinations of neural networks with underlying physics, as well as the challenges and limitations of DL in inverse scattering. A survey focused on ML in antenna design and optimization [2] examined regression models as well as ML-based antenna synthesis and analysis. A broader review [3] surveyed ML applications across electromagnetics, including antenna design, inverse scattering, radar, sensing, and fault detection. In the optimization domain, a comprehensive survey focused on EM-based optimization algorithms [4] classified methods into direct and surrogate optimization, including surrogate model and artificial neural network (ANN)-based optimization examples for transmission lines, filters, and antennas. More recently, a review of artificial intelligence (AI)/ML in antenna design optimization and measurement [5] examined recent AI/ML approaches for antenna-related applications and discussed the associated challenges, limitations, and future research opportunities. Sendrea et al. [6] provided a comprehensive review of multifidelity learning approaches for EM problems, covering forward and inverse problems, low-fidelity data generation, and physics-based learning approaches. The above reviews address important but different aspects of inverse scattering, antenna-focused ML, broad EM applications, optimization methods, and multifidelity modeling. However, according to their stated scope, they do not clearly present a workflow synthesis specifically focused on surrogate-assisted EM-driven design and optimization of RF and microwave components.

The above review articles were used only for contextual positioning and comparative framing in Table 1. They were not part of the PRISMA-coded Scopus screening corpus used for the formal evidence synthesis presented in this review.

Table 1 shows that recent reviews are clearly valuable, but they address different and usually narrower scopes compared with this review. Some of them have mainly focused on antenna design, whereas others have focused on inverse scattering, optimization methods, or multifidelity learning. Broader reviews have discussed ML in electromagnetics at a general level. In contrast, this review is positioned as a systematic review of surrogate-assisted EM-driven design and optimization across RF/microwave component classes, where the design workflow is the main scope of our analysis. Therefore, it does not replace earlier reviews in their specialized domains; instead, it complements them by providing a unified workflow-based synthesis of using surrogate models in RF/microwave design practice across different component categories. Additionally, it examines their role in forward and inverse design, their integration with multifidelity strategies, the adoption of active learning, and the presence of fully integrated pipelines. Such a workflow-based synthesis has not been reported in previous reviews. This gap provides the motivation for this review and directly supports the research questions (RQs) stated in the next subsection.

### 1.2. Review Scope and Research Questions

This review examines the integration of surrogate models into EM-driven design workflows, their role in forward and inverse design tasks, the integration of multifidelity strategies, the adoption of active learning or adaptive sampling, and the existence of unified workflows that combine these capabilities. Applications outside the RF and microwave component design workflows (such as biomedical systems, electric motors, thermal modeling, computational fluid dynamics (CFDs)-only applications, and other non-EM domains) have been excluded from the scope of this review. The evidence base was constructed by conducting a structured Scopus search. The detailed search strategy, screening process, and eligibility criteria are described in Section 2.

Accordingly, our review was guided by the following RQs:

1. **Role of Surrogate Models in RF/Microwave Design Workflows:** How are surrogate models functionally positioned within RF and microwave design workflows?
2. **Inverse-Design Adoption:** To what extent are surrogate models used in inverse RF/microwave design, either through explicit inverse mapping or optimization-based inverse design?
3. **MultiFidelity Integration:** How frequently are multifidelity or variable-fidelity EM strategies integrated into surrogate-based RF/microwave design and optimization workflows?
4. **Active Learning Adoption:** To what extent is active learning or adaptive sampling systematically incorporated into RF and microwave surrogate modeling workflows?
5. **Unified Workflow:** Do existing studies implement a unified workflow that combines surrogate modeling, inverse design, multifidelity interaction, and active learning within a unified RF/microwave design framework?

To address the above RQs, we combined PRISMA-guided study-screening with a structured workflow-based classification process and a descriptive comparative synthesis. The methodological procedure used for identifying, screening, classifying, and analyzing the existing studies is presented in the next section.

## 2. Materials and Methods

This section describes the search strategy, study selection process, eligibility criteria, and coding framework used in the review. The methodology was designed to ensure consistent inclusion decisions and transparent classification of surrogate-assisted RF and microwave design studies.

### 2.1. Search Strategy and Study Identification

A structured literature search was conducted in the Scopus database (Elsevier, Amsterdam, The Netherlands) to identify journal articles related to surrogate-assisted RF and microwave design. The search targeted studies published between 2012 and February 2026 and was restricted to English-language journal articles indexed in the Engineering and Computer Science subject areas. Only open access research articles were considered. The full Scopus search query used in this review is provided in Box 1.

Box 1 **Full Scopus search query used in this review.**

TITLE-ABS-KEY (“surrogate model” OR “surrogate modeling” OR “surrogate modelling” OR “machine learning surrogate” OR “metamodel” OR “kriging” OR “Gaussian process” OR “neural network surrogate”) AND TITLE-ABS-KEY (“microwave circuit” OR “microwave” OR “RF circuit” OR “RF component” OR “microwave component” OR “passive component” OR “electromagnetic simulation” OR “EM design” OR “computational electromagnetics” OR “full-wave simulation”) AND PUBYEAR > 2011 AND PUBYEAR < 2027 AND (LIMIT-TO (SRCTYPE, “j”)) AND (LIMIT-TO (OA, “all”)) AND (LIMIT-TO (DOCTYPE, “ar”)) AND (LIMIT-TO (SUBJAREA, “ENGI”) OR LIMIT-TO (SUBJAREA, “COMP”)) AND (LIMIT-TO (LANGUAGE, “English”))

The study selection process, eligibility filtering, and final corpus definition are described in the following subsections.

### 2.2. Eligibility Criteria

The eligibility of a study was determined using a workflow-based screening logic rather than a contribution-based one. Accordingly, each study was classified not only by its stated methodological contribution but also by the design-workflow mechanism it implemented. Classification was assigned only when the workflow element was clearly implemented and supported by meaningful design results. When a workflow element was unclear or insufficiently supported, a more conservative classification was adopted. A study was included in this review only if it satisfied all the following criteria: (1) The study addressed a solid RF/microwave component case or a clearly identifiable RF/microwave functional structure; eligible examples included antennas or reflectarray antennas, filters, couplers, dividers, resonators, matching networks, impedance transformers, power and low-noise amplifiers, rectifiers, sensors, rectennas, metasurfaces, and frequency-selective-surface unit cells. (2) The study contained a surrogate, regression, or approximation model of the EM response or the model replaced part of the EM evaluation process; examples include Gaussian process or Kriging models, co-Kriging models, neural networks, support vector regression, polynomial or response-surface models, relevance vector regression, polynomial chaos expansion, and related regression-based surrogate formulations. (3) The surrogate model was crucial in the RF/microwave design process; for example, in forward prediction, optimization, inverse design, uncertainty or sensitivity analysis, or within a multifidelity framework.

Additionally, applications that were not related to RF/microwave component design workflows, such as biomedical systems, electric motors, thermal modeling, CFD-only applications, or other non-EM domains, were excluded. Furthermore, studies focused only on generic numerical solvers, general optimization methods, or ML demonstrations without a solid RF/microwave component workflow were considered out of scope.

### 2.3. Study Selection Process and Final Review Corpus

**Figure 1:**
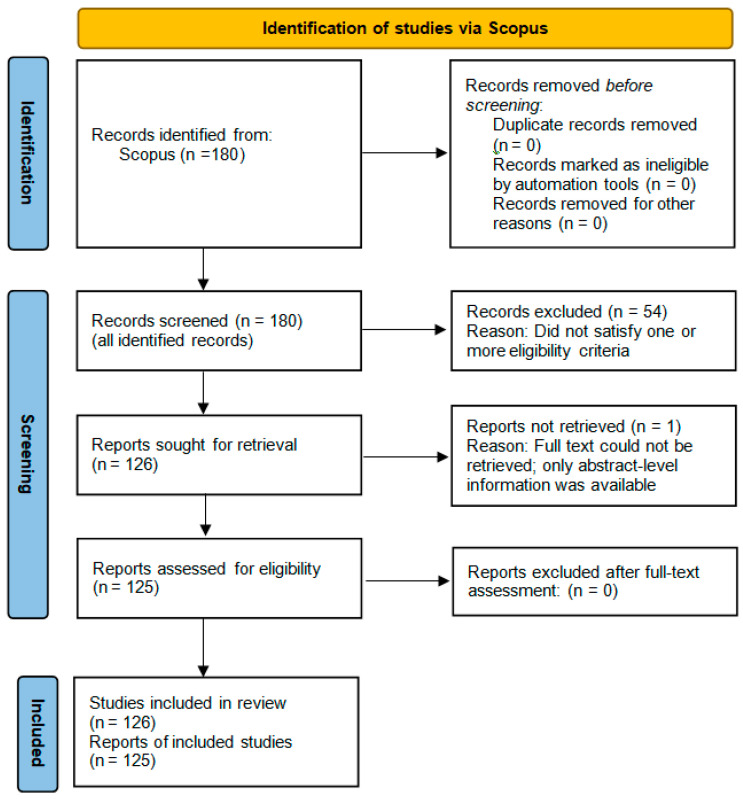
PRISMA-guided study-selection and eligibility-filtering process of the Scopus-derived literature corpus.

The study selection process (Figure 1) was based on the PRISMA-inspired screening principles. The Scopus search identified 180 records. After title, abstract, and full text screening, 54 records were excluded because they did not meet one or more eligibility criteria, and 126 studies [7,8,9,10,11,12,13,14,15,16,17,18,19,20,21,22,23,24,25,26,27,28,29,30,31,32,33,34,35,36,37,38,39,40,41,42,43,44,45,46,47,48,49,50,51,52,53,54,55,56,57,58,59,60,61,62,63,64,65,66,67,68,69,70,71,72,73,74,75,76,77,78,79,80,81,82,83,84,85,86,87,88,89,90,91,92,93,94,95,96,97,98,99,100,101,102,103,104,105,106,107,108,109,110,111,112,113,114,115,116,117,118,119,120,121,122,123,124,125,126,127,128,129,130,131,132] were included in the final review corpus.

In one of these studies, only abstract-level information was available because the full text could not be retrieved. Considering that the available abstract still provided sufficient evidence that all three eligibility criteria were satisfied, the study was included in the corpus. However, it was not entered into the detailed technical classification, taxonomy, or gap analysis tables because full-text evidence was not available; instead, it was used only in limited descriptive elements that could be supported by the available information such as source-level identification and year-based summaries.

Accordingly, the total included literature consisted of 126 studies; the detailed technical classification, taxonomy assignment, and comparative synthesis were based on 125 fully classifiable studies. The six review articles discussed in Section 1 were used separately for contextual positioning and comparative framing; they were not part of the screened main-study corpus.

Figure 1 shows the overall study-selection and eligibility-filtering process for the Scopus-derived literature corpus.

### 2.4. Classification Principles

This section describes the classification framework used for classifying the included studies, the workflow-based and technical descriptors for data extraction, the operational rules for assigning the main classification fields, and the decision process for deriving the final taxonomy categories across the review corpus.

#### 2.4.1. Mapping and Technical Classification Fields

For each study included in this review, the information was organized into two structured classification tables. The first table includes the main workflow-based classification fields used in this review, such as publication year, dominant RF/microwave component type, passive or active role, surrogate family, and inverse approach, as well as design-optimization, multifidelity, and active-learning statuses. When more than one structure or application example was reported in a study, the component label was assigned according to the dominant RF/microwave design target of the surrogate-participating workflow. This was especially relevant in studies where multiple structures were discussed, but ultimately the focus was on one main design target such as an antenna, filter, coupler, impedance transformer, or amplifier [8,13,57].

The second layer was a technical classification table that was used to capture more detailed workflow characteristics, including differentiability, degree of physics-informed modeling, surrogate usage mode, multifidelity and active-learning strategies, optimizer family, fabrication status, and EM solver environment. Both tables were used to support the later synthesis of workflow patterns, the gap-oriented analysis, and the final taxonomy assignment. The mapping, technical, taxonomy, and gap-oriented tables, along with the graphical summaries of the included studies, are provided in Supplementary File S1. Ref. [47] was not included in the detailed tables because the full text of that study was not available.

#### 2.4.2. Decision Rules for Mapping Table Fields

The main workflow attributes in the mapping table were assigned according to predefined decision rules, strictly based on the reported workflow of each study included in this review. These rules were used to align the classification with the actual design process implemented in each study rather than with the broader methodological framing used by the authors.

Initially, the inverse-design-approach field was assigned only when the workflow was clearly specification-driven. Existing-design tuning or generic performance improvements were not considered inverse-design approaches. This distinction was particularly important in antenna studies, where the geometry was optimized to improve bandwidth, gain, and reflection performance without explicitly defining electrical parameter target specifications before the search began. Accordingly, the inverse-design approach was determined using a four-step decision process. If a study contained a learned mapping from specifications to geometry, it was classified as explicit inverse when no optimization refinement was used, or as a hybrid inverse framework when the inverse model was followed by, or used together with, optimization refinement. If no direct inverse mapping was present, the study was checked for explicit electrical parameter target specifications that were defined before optimization. Only when such predefined specifications were present and the parameter–space search was performed on the design variables to satisfy the specifications, the study was classified as optimization-based inverse; otherwise, the inverse-design approach was classified as “none”. Based on this rule, a general performance-driven optimization without predefined target specifications was classified as a forward optimization rather than an inverse-design approach. Representative examples of explicit inverse workflows were identified in [57,92,105,115]; hybrid inverse workflows were identified in Refs. [40,49,58,114]. In contrast, Refs. [28,61] were classified as “non-inverse” because they reported surrogate-assisted optimization without predefined target specifications.

Next, design optimization was recorded only when the study reported an actual parameter–space search on the design variables and produced meaningful design results. Such studies included surrogate-assisted optimization loops and workflows in which the final search relied directly on EM-based evaluations. Pure forward prediction or modeling studies without a validated search stage were not classified as optimization studies. Similarly, schematic or purely illustrative optimization examples without a substantive design outcome were not treated as optimization cases.

Finally, multifidelity and active learning were recorded as binary workflow attributes at the mapping-table level. Multifidelity was recorded only when at least two distinct model fidelities interacted within the workflow. Active learning was recorded only when the study reported an explicit policy for selecting new samples. A detailed technical characterization of these attributes, including specific multifidelity and active-learning strategies, was performed separately in the technical classification table, as described in the following subsection.

#### 2.4.3. Decision Rules for Technical Classification Fields

The technical classification table was used to describe how the surrogate model functioned within the implemented workflow and to record additional methodological characteristics that were not fully represented by the mapping table alone. These fields were assigned according to explicit interpretation rules to consistently describe technically heterogeneous studies in the review corpus.

Differentiability was recorded only when the study explicitly reported or clearly described an end-to-end differentiable workflow with respect to the design variables. When differentiability was not explicitly stated or could not be verified using the reported workflow, the study was conservatively coded as “non-differentiable.” This distinction was most relevant in differentiable-inverse or refinement-oriented surrogate workflows, whereas the Gaussian process, Kriging models, support-vector-regression, and other black-box surrogate implementations were not coded as “differentiable.”

The degree of physics-informed modeling was classified as “No,” “Partial,” or “Full.” Full physics-informed modeling was reserved for studies that embedded explicit physical equations or constraints directly into training. Partial physics-informed modeling was assigned when explicit physical knowledge was used to support the surrogate construction without becoming a formal governing-equation constraint; for example, via equivalent-circuit priors, coarse-model guidance, space-mapping structures, or physically meaningful engineered response features. Purely data-driven regression obtained from sampled responses was coded as non-physics-informed. Importantly, the use of EM-generated data alone was not considered sufficient to classify a study as physics-informed. Representative partially physics-informed workflows were included in Refs. [26,59,122].

The surrogate usage mode was not assigned independently; instead, it was directly derived from the combination of the inverse-design approach and design optimization. Studies classified as **explicit inverse**, **optimization-based inverse**, and **hybrid inverse frameworks** were mapped directly to the corresponding surrogate-usage modes. When an inverse-design approach was recorded as “**none**”, studies with design optimization were coded as **surrogate-assisted optimization**, whereas studies without design optimization were coded as **forward-design only**. This rule was used to keep the technical characterization consistent with the mapping-table decisions.

The multifidelity strategy field was assigned only to studies already coded as “multifidelity” at the mapping-table level; otherwise, it was recorded as “none.” In these studies, the specific multifidelity strategy was recorded as co-Kriging coupling, space mapping, trust-region-based multifidelity management, hierarchical surrogate structure across distinct fidelity levels, physics-based correction, or more generic low and high-fidelity correction, depending on the mechanism explicitly implemented in the reported workflow. Similarly, the active-learning strategy field was assigned only to studies already coded as active learning; otherwise, it was recorded as “none.”

The optimizer type was assigned according to the optimizer family used in the final design-search workflow rather than according to methods mentioned only in the discussion or previous literature. Evolutionary optimization included population-based metaheuristics such as genetic algorithms, particle swarm optimization, differential evolution, and related variants. Gradient-based optimization included explicitly gradient-driven or quasi-Newton-type searches, whereas deterministic derivative-free optimization included direct-search and pattern-search methods. Bayesian optimization was assigned only when the workflow explicitly used Bayesian optimization or acquisition-function-based search. Hybrid optimization was assigned only when two optimizer families were crucial within the same integrated optimization framework or design-search loop. Space-mapping-based optimization and sequential domain patching were also treated as distinct technical categories when explicitly implemented; named optimizers that could not be mapped to the predefined categories were assigned to “other.” When optimization was clearly present, but the optimizer was not explicitly identified, the field was coded as “Not mentioned.” The optimizer type was treated as a technical descriptor of the implemented search mechanism and not as a direct proxy for the later taxonomy assignment.

Additionally, the technical table recorded the fabrication status and EM solver environment. A study was marked as “Fabricated and Measured only” when a physical prototype and measurement results were reported; otherwise, the study was coded as “simulation-only.” The EM-solver field recorded the software environment, such as HFSS, CST, ADS, Sonnet, COMSOL, FEKO, or another named solver, that was explicitly reported in the study. These fields were included to support the later interpretation of validation depth and implementation context across the literature included in this review.

#### 2.4.4. Consistency Rules and Derived Fields

To preserve internal consistency across the classification framework, several fields were constrained by setting explicit dependency rules rather than being treated as fully independent coding decisions. This ensured that the mapping table, the technical classification table, and the later summary analyses remained structurally aligned throughout the review.

Certain technical fields were recorded only when the corresponding higher-level workflow attribute had already been verified. Specifically, the multifidelity and active-learning strategies were assigned only to studies already coded as “multifidelity” and “active learning,” respectively; otherwise, these fields were recorded as “none.” Similarly, the optimizer type was recorded only for those studies where design optimization had been verified. These dependency rules were adopted to prevent technical subcategories from being assigned in the absence of the underlying workflow mechanism.

Furthermore, the binary indicators that were later used in the gap-oriented synthesis were derived directly from the coded workflow fields rather than being separately assigned. The surrogate indicator was implicitly positive for the full corpus because all studies included in this review already satisfied the surrogate-related inclusion criteria. The inverse indicator was assigned positively whenever the inverse-design approach field was different from “none.” The multifidelity and active-learning indicators followed the verified multifidelity field and the corresponding active-learning field, respectively, in the mapping table. In this way, the gap-oriented summary table was mechanically derived from the master classification sheet rather than from separate manual counting.

These dependency rules were collectively used to preserve consistency among workflow-level coding, technical characterization, and summary-level synthesis. Additionally, they reduced the risk of internal contradictions between related fields and ensured that later comparative analyses remained directly traceable to the original study-level classifications.

#### 2.4.5. Taxonomy Assignment

After completing the mapping and technical classification tables, each fully classifiable study was assigned to one taxonomy category. As previously noted in Section 2.4.1, Ref. [47] was excluded from this step because full-text evidence was not available. The taxonomy was designed as a mutually exclusive workflow framework. Its purpose was to capture the dominant workflow architecture of each study rather than every mechanism that coexisted within the same paper. Considering that many studies contain multiple workflow characteristics, the taxonomy assignment followed a fixed decision order rather than a one-to-one mapping from a single field.

The taxonomy was applied according to the following priority order: unified integrated pipelines, active-learning EM frameworks, multifidelity surrogate frameworks, surrogate-assisted evolutionary optimization, hybrid optimization frameworks, surrogate-assisted optimization, inverse surrogate models, and forward EM surrogate models. The taxonomy category “unified integrated pipelines” was used only as a formal category within the taxonomy framework. It was considered separately from the unified-workflow condition examined in RQ 5, which specifically referred to the simultaneous presence of surrogate modeling, inverse design, multifidelity interaction, and active learning within the same study. Under this rule, once a study satisfied a high-priority category, it was not further considered for low-priority categories. This was particularly important in studies where active learning or multifidelity interaction coexisted with optimization or inverse-design elements [72,114].

Accordingly, the taxonomy assignment was not determined considering only the inverse-design approach; additionally, it was not identical to optimizer-family coding. The optimizer type recorded the optimizer family that was actually used in the implemented design-search workflow, whereas the taxonomy recorded the dominant workflow category after the full decision order had been applied. For example, a study on a space-mapping-based optimizer could still be assigned to multifidelity surrogate frameworks when multifidelity interaction had higher priority in the taxonomy hierarchy [26]. Similarly, a study reporting Bayesian optimization could still be assigned to active-learning EM frameworks when active learning was present, because of the higher priority in the taxonomy decision order [72]. Additionally, the surrogate usage mode was not equivalent to taxonomy: a study on the hybrid inverse framework surrogate usage could still be assigned to multifidelity surrogate frameworks when the priority of multifidelity interaction was higher [114] or to surrogate-assisted optimization when the taxonomy decision order reached the corresponding optimization category before the inverse surrogate level [40].

Furthermore, surrogate-assisted evolutionary optimization was assigned only when design optimization was present, the optimizer type was classified as evolutionary, and no higher-priority active-learning or multifidelity category had already been assigned. Hybrid optimization frameworks were assigned only when the optimizer type was classified as hybrid and no higher-priority category had already been assigned. Then, surrogate-assisted optimization covered the remaining optimization studies using deterministic, gradient-based, Bayesian optimization, space mapping-based, semidefinite programming (SDP), other, or not mentioned optimizer families, again only when no higher-priority category had already been assigned. Therefore, the inverse surrogate model category was assigned only after all higher-priority active-learning, multifidelity, and optimization categories had already been checked. This category was reserved for studies with a surrogate usage mode that was explicit-inverse surrogate usage mode that was explicit-inverse surrogate, optimization-based inverse design, or hybrid inverse frameworks that had not already been assigned to any earlier category. Finally, forward EM surrogates served as the residual category for studies without design optimization, with a forward-only surrogate usage mode.

This taxonomy procedure was separate from the binary gap-oriented synthesis. The gap table was derived from the coded workflow fields in the mapping table and their associated binary derivations, whereas the taxonomy table was derived from the fixed decision order applied after mapping and technical classification had already been completed. In this way, the gap analysis summarized the coexistence of surrogate, inverse, multifidelity, and active-learning attributes and the taxonomy assigned one dominant workflow category to each fully classifiable study.

#### 2.4.6. Assessment of Reporting Sufficiency and Classification Consistency

An additional structured assessment was used to support consistent interpretation and classification across the final review corpus because the studies included in this review varied considerably regarding their RF/microwave design targets, surrogate strategies, and workflow structures. This assessment was not used to determine whether a study should be included or excluded. Instead, it was used to examine whether each study was reported clearly enough to support the workflow-based classification framework adopted in this review.

The purpose of this assessment was to determine whether each study provided enough explicit information to support the main classification decisions used in the review. Practically, the assessment focused on the following main questions:

1. Were the RF/microwave design target and application context described clearly enough to support eligibility and scope classification?
2. Were the surrogate model and its functional role in the workflow described clearly enough to support mapping-table classification?
3. Was the methodological workflow described clearly enough to support the classification of design optimization, inverse-design approach, multifidelity interaction, and active learning?
4. Were the reported methods and results sufficiently clear to support technical characterization, taxonomy assignment, and later cross-study synthesis?

This assessment was not intended to produce a numerical quality score and was not used to assess studies in terms of their overall methodological quality. Instead, it served as a structured support step for classification. When reporting was incomplete, unclear, or not sufficiently explicit, only the attributes directly supported by the text were assigned, and the more conservative valid category was selected. In this way, the assessment supported the rule-based classification logic of this review without introducing a separate quality-scoring system.

Formal agreement statistics were not calculated. Instead, consistency was supported by predefined field definitions, conservative classification rules, and repeated cross-checking across the mapping table, the technical classification table, the derived binary indicators, and the final taxonomy assignment.

A formal risk-of-bias checklist was not used in this review. Instead, the review used a structured assessment of reporting sufficiency to support consistent classification, as this was more closely aligned with the workflow-based classification objectives of the study.

As previously noted in Section 2.4.1, Ref. [47] was not included in the detailed technical classification, taxonomy, or gap analysis tables due to the absence of full text.

The complete coded evidence base is documented in Supplementary File S1.

## 3. Results

In this section, we present the results of this review according to the main analytical dimensions defined in the methodology. Initially, we present an overview of the studies included in this review, and then we present the main classification results related to surrogate families, inverse design, multifidelity and active learning, workflow feature combinations, functional taxonomy, and reporting quality.

### 3.1. Overview of the Included Studies

The final review corpus consisted of 126 studies, which were identified using the Scopus-based screening process described in Section 2. A total of 125 of these studies were fully classifiable at the detailed workflow level and were used in the formal technical classification, taxonomy assignment, and gap-oriented synthesis, excluding [47] (as described in Section 2.4.1).

**Figure 2:**
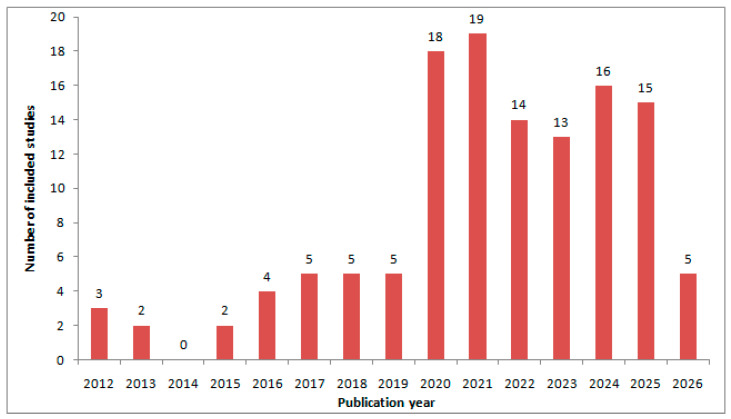
Annual distribution of the included studies published between 2012 and February 2026.

Figure 2 shows the temporal distribution of the studies included in this review during the period from 2012 to February 2026. The relevant literature was limited in the early years of the review window (only a few studies were published between 2012 and 2015). A gradual increase became visible in 2016, followed by a clear increase after 2019. The strongest publication activity was observed during the 2020–2025 period; the yearly counts were consistently higher in the earlier years. The highest yearly count was recorded in 2021, followed by similarly strong activities in 2024 and 2025. Overall, this pattern indicates that surrogate-assisted RF/microwave design has developed from a relatively specialized topic into a more established and actively growing research area.

**Figure 3:**
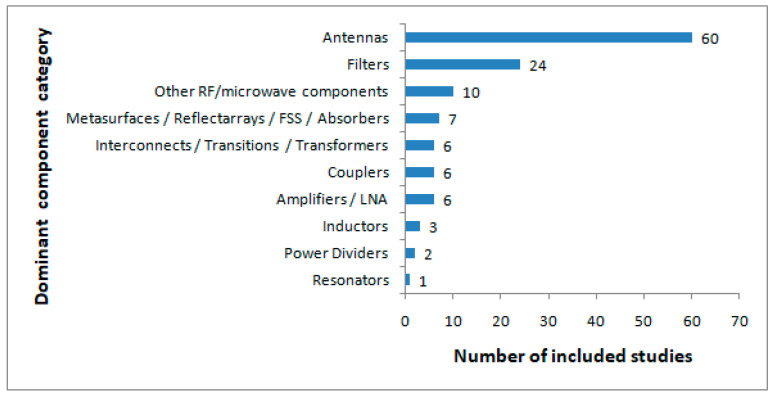
Distribution of the dominant RF/microwave component categories across the fully classifiable studies.

In terms of component distribution, the included literature was clearly dominated by antenna-related studies, followed by filter design; all other RF and microwave component categories appeared much less frequently. As shown in Figure 3, studies on antennas and filters were reported in 60 and 24 articles, respectively; the remaining literature was distributed across smaller categories, including couplers, interconnects or impedance transformers, amplifiers or low-noise amplifiers, metasurfaces or reflectarray-related structures, and other less frequently presented RF/microwave component types. The broader “other RF/microwave components” category included a small number of more diverse cases, such as vircators, traveling-wave tubes, dielectric waveguide structures, shielding structures, transition-related implementations, and general microwave-device studies that did not clearly fit into the main component groups. This distribution shows that surrogate-assisted design has been most often used in component classes where EM simulations were computationally demanding, and repeated design evaluation was common.

Collectively, these descriptive results show that the evidence base is both recent and concentrated in a small number of dominant component categories. Most of the included studies were published in the later years of the review period, and a large share of the literature focused on antennas and filters rather than being evenly distributed across RF/microwave component classes. This broad overview sets the general background for the more detailed workflow-based results presented in the following subsections.

### 3.2. Surrogate Families and Workflow Roles

This subsection addresses RQ 1, which examines how surrogate models are functionally positioned within RF/microwave design workflows. To answer this question, we examined both the main surrogate model families used across the studies included in this review and how these surrogate models are integrated into the implemented workflow.

**Figure 4:**
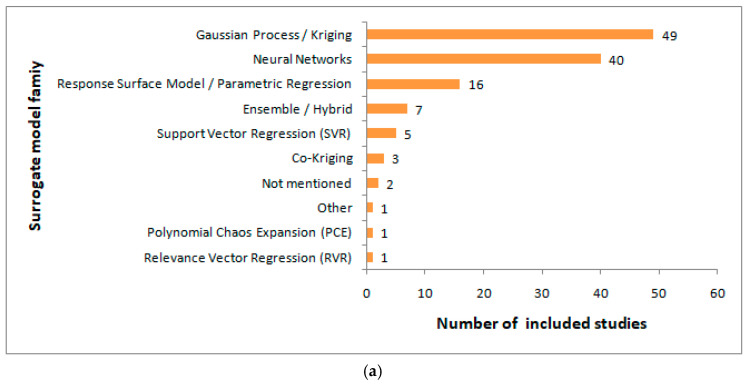

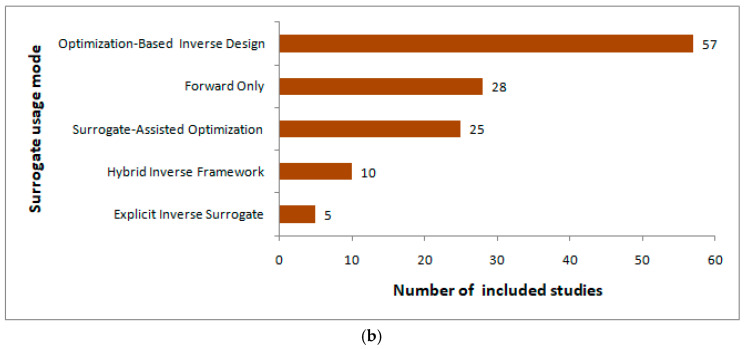
Surrogate model-related distributions across the fully classifiable studies: (**a**) surrogate model family; (**b**) surrogate model usage.

Figure 4a shows the distribution of surrogate model families across the fully classifiable studies. The most frequently reported surrogate family was the Gaussian process or Kriging models (49 studies), followed by neural networks (40 studies). Collectively, these two families accounted for most of the reviewed literature. More traditional regression-based approaches, which were grouped here as response surface models or parametric regression, appeared in 16 studies. All other surrogate families, including ensemble or hybrid models, support vector regression, co-Kriging, polynomial chaos expansion, relevance vector regression, and a few cases classified as “other” or “not mentioned,” were much less frequent.

This distribution shows that the literature has largely focused on two main surrogate families. The Gaussian process and Kriging methods were especially prominent across the reviewed literature and continue to appear as a common surrogate choice in RF/microwave design studies. Neural networks also appear often, indicating that data-driven surrogate formulations are now a major part of this research area alongside more established regression-based approaches. In contrast, the smaller presence of the remaining surrogate families indicates that they are used more selectively and usually in more specific methodological settings.

Figure 4b shows how surrogate models are functionally positioned within the implemented RF/microwave design workflows. The most common use of surrogate models is the optimization-based inverse design (57 studies), followed by forward-only workflows (28 studies) and surrogate-assisted optimization (25 studies). Less frequent categories include hybrid inverse frameworks (10 studies) and explicit inverse surrogate models (5 studies).

These results indicate that surrogate models are mostly used as part of target-driven design workflows rather than only as forward predictors. The dominance of optimization-based inverse design indicates that, in many studies, surrogate models were not used simply to approximate EM responses but to support the search for design parameters that satisfy predefined electrical specifications. The presence of 28 forward-only studies indicates that surrogate models have been reported in a substantial part of the literature, mainly for response prediction, modeling, or performance estimation, without a full design-search stage. The 25 studies classified as surrogate-assisted optimization further show that many workflows rely on surrogate models to improve the efficiency of design refinement or parameter search, even when the workflow is not formally treated as inverse design under the strict classification rules adopted in this review.

The small number of studies on explicit inverse surrogate models and hybrid inverse frameworks is also informative. Fully learned direct inverse mappings from specifications to geometry are relatively uncommon. Hybrid workflows combining inverse prediction with subsequent refinement are present but still limited compared with optimization-based inverse design. Overall, the results indicate that surrogate models in RF/microwave design are mostly used as embedded workflow components within broad optimization or specification-driven design procedures rather than as standalone forward prediction tools.

Collectively, the results presented in this subsection answer RQ 1, indicating that surrogate models are functionally positioned in several distinct ways, but most often in workflows, where they support inverse design, design search, or specification-driven parameter selection. Practically, the relevant literature is dominated by a combination of two main surrogate families (Gaussian process or Kriging models and neural networks) and by workflow roles where surrogate models support inverse design or search-based design refinement rather than forward EM-prediction only.

### 3.3. Adoption of Inverse Design

This subsection addresses RQ 2, which examines the extent to which surrogate models are used in inverse RF/microwave design, either via explicit inverse mapping or optimization-based inverse design. In this review, inverse design was classified conservatively and recorded only when the workflow was explicitly specification-driven, according to the decision rules defined in Section 2.

**Figure 5:**
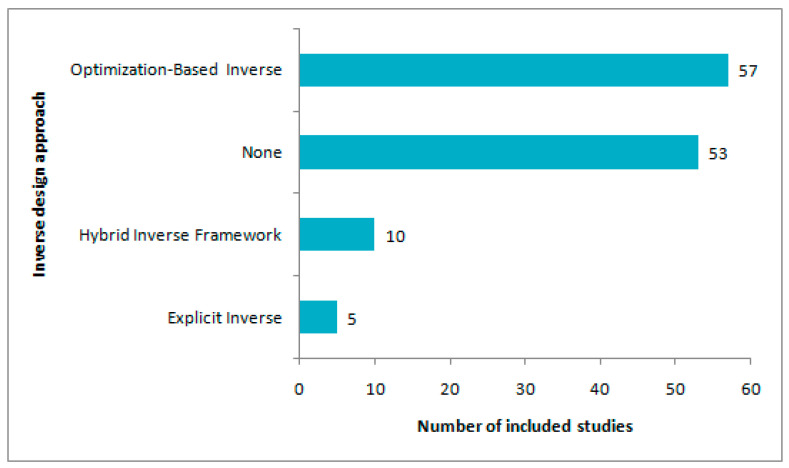
Distribution of inverse design approaches across the fully classifiable studies.

Figure 5 shows the distribution of inverse-design approaches across the fully classifiable studies. The most frequent category was optimization-based inverse design, which appeared in 57 studies, followed by 53 studies classified as “none.” Less frequent categories included hybrid inverse frameworks (10 studies) and explicit inverse approaches (5 studies).

These results show that inverse design was common in the reviewed literature; however, it is not reported in all surrogate-based workflows. Considering all inverse categories, 72 of the 125 fully classifiable studies were classified as inverse-design studies, whereas 53 were classified as non-inverse under the strict workflow-based definition used in this review. Therefore, inverse design represents a substantial part of the reviewed literature, although many surrogate-based workflows are outside the inverse-design category under the adopted classification rules.

This distribution clearly shows the predominance of optimization-based inverse design, indicating that the most common inverse-design implementation in the reviewed literature is not a direct learned mapping from specifications to geometry, but a specification-driven search process, where surrogate models support the optimization of design variables toward predefined electrical parameter targets. In contrast, explicit inverse approaches were relatively uncommon (5 studies). Hybrid inverse frameworks, which combine inverse prediction with subsequent refinement, were more frequent than fully explicit inverse models but much less common than optimization-based inverse workflows.

Collectively, the results presented in this subsection answer RQ 2, indicating that inverse design has been well reported in the reviewed literature; however, it is mostly implemented via optimization-based inverse workflows rather than explicit inverse surrogate mappings. Optimization-based inverse implementations were far more common than direct explicit inverse mappings in the studies classified as inverse-design workflows.

### 3.4. Adoption of Multifidelity and Active Learning

This subsection addresses RQs 3 and 4, which examine how frequently multifidelity strategies are integrated into surrogate-based RF/microwave design workflows and to what extent active learning or adaptive sampling is systematically incorporated.

**Figure 6:**
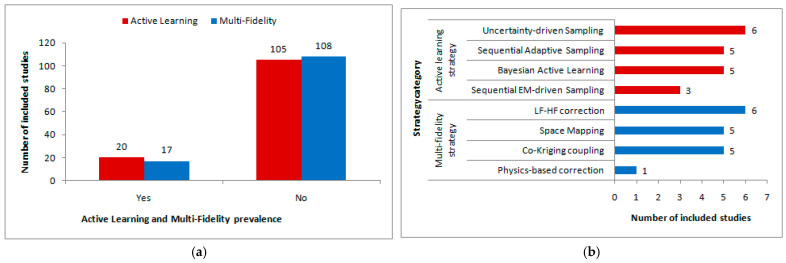
Adoption of active learning and multifidelity across the fully classifiable studies: (**a**) prevalence of active learning and multifidelity; (**b**) strategy categories in the studies classified as active learning or multifidelity. Red indicates active learning, while blue indicates multi-fidelity.

Figure 6a shows the prevalence of active learning and multifidelity across the fully classifiable studies. Active learning was identified in 20 studies and was absent in 105, whereas multifidelity was identified in 17 studies and was absent in 108. In both cases, most of the reviewed literature did not include these workflow elements.

These results indicate that both active learning and multifidelity have been reported in the reviewed literature; however, neither has been widely adopted across the full corpus. Active learning was reported more often than multifidelity, although the difference was small. Overall, both workflow features were clearly less common than their absence, indicating that most surrogate-based RF/microwave studies still rely on simple workflows without explicit adaptive sample selection or multilevel fidelity interaction.

Figure 6b further shows a breakdown of the specific strategy categories used in the studies that were classified as active learning or multifidelity. Among the active-learning studies, the most frequent strategy was uncertainty-driven sampling (six studies), followed by sequential adaptive sampling and Bayesian active learning (five studies each), and sequential EM-driven sampling (three studies). Among the multifidelity studies, the most frequent strategy was low-frequency (LF)/high-frequency (HF) correction (six studies), followed by space mapping and Co-Kriging coupling (five studies each); physics-based correction appeared in only one study.

These results show that neither active learning nor multifidelity is concentrated around one clearly dominant implementation. Regarding active learning, uncertainty-driven sampling was the most frequent category, but only by a small margin over sequential adaptive sampling and Bayesian active learning. Regarding multifidelity, LF/HF correction, space mapping, and co-Kriging coupling appeared at similar levels, and explicit physics-based correction was scarce.

In summary, the results presented in this subsection answer RQs 3 and 4, indicating that the adoption of both multifidelity and active learning is still limited across surrogate-assisted RF/microwave design studies. When these workflow elements do appear, they are implemented via several methodological strategies rather than a single clearly dominant approach.

### 3.5. Workflow Feature Combinations and the Unified-Workflow Question

This subsection addresses RQ 5, which examines whether the reviewed literature contains studies that have implemented a unified workflow combining surrogate modeling, inverse design, multifidelity interaction, and active learning within the same RF/microwave design framework. To answer this question, this review considers the binary coexistence of these workflow features across the fully classifiable studies rather than the separate taxonomy categories presented earlier.

**Figure 7:**
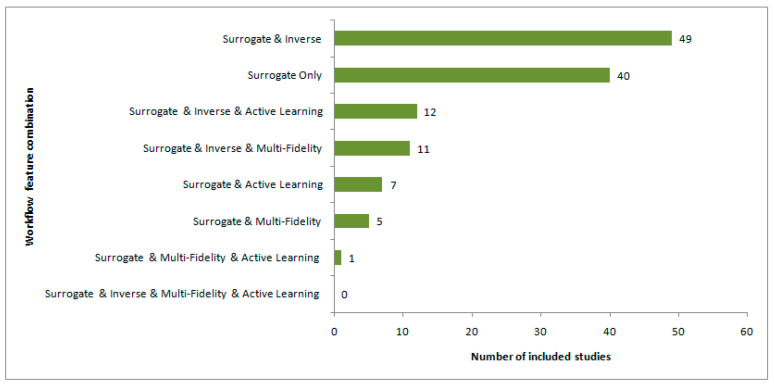
Distribution of workflow feature combinations across the fully classifiable studies, considering the coexistence of surrogate modeling, inverse design, multifidelity interaction, and active learning.

Figure 7 shows the observed combinations of workflow features across the reviewed corpus. The most frequent combination was surrogate and inverse (49 studies), followed by surrogate only (40 studies). Additional recurring combinations included surrogate and inverse and active learning (12 studies), and surrogate and inverse and multifidelity (11 studies). Less frequent combinations were surrogate and active learning (seven studies), surrogate and multifidelity (five studies), and surrogate and multifidelity and active learning (one study). Interestingly, the combination of surrogate and inverse and multifidelity and active learning did not appear in any fully classifiable study.

These results indicate that partial combinations of workflow features are present in the reviewed literature; however, the full four-feature combination required by RQ 5 was not observed. Practically, many studies combined surrogate modeling with inverse design, whereas a few studies combined surrogate modeling with either active learning or multifidelity interaction. Some studies combined surrogate modeling, inverse design, and one additional workflow element, either active learning or multifidelity. However, no study in the fully classifiable corpus has simultaneously combined all four elements within the same implemented workflow.

This distinction is important because the workflow combination analysis used in this subsection is not identical to the taxonomy assignment presented earlier. In particular, the taxonomy category “unified integrated pipelines” was a formal workflow category assigned according to the taxonomy decision order described in Section 2.4.5. In contrast, the unified-workflow condition examined here refers specifically to the explicit coexistence of all four workflow features in the same study. Consequently, a study could be assigned to the above taxonomy category without satisfying the four-feature condition used here.

Accordingly, the results presented in this subsection indicate that the reviewed literature contains several partially integrated workflow patterns but no fully unified workflow under the definition adopted in this review. This directly answers RQ 5.

### 3.6. Functional Workflow Taxonomy of the Studies Included in This Review

**Figure 8:**
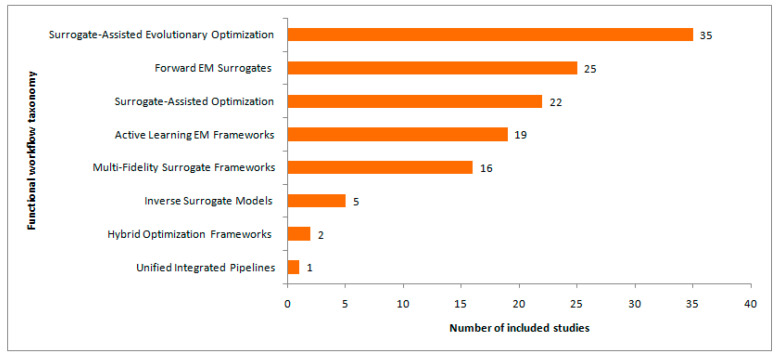
Distribution of the final functional workflow taxonomy categories across the fully classifiable studies based on the mutually exclusive decision hierarchy described in Section 2.4.5. Each study was assigned to one dominant category only.

This subsection summarizes the final functional workflow taxonomy across the fully classifiable studies. Unlike the feature-combination analysis presented in the previous subsection, the taxonomy assigns each study to one and only one dominant workflow category according to the fixed decision order described in Section 2.4.5. Therefore, the purpose of this taxonomy is not to show the coexistence of multiple workflow features within the same study but to provide a mutually exclusive classification of the dominant workflow structure across the studies included in this survey. The taxonomy categories and their operational meaning are summarized in Table 2. Figure 8 shows their distribution across the included studies. Ref. [47] was retained in the broader review corpus; however, for the reasons discussed in Section 2.4.1, it was not included in the formal taxonomy assignment. Therefore, the taxonomy presented here is based on 125 fully classifiable studies.

Overall, the taxonomy shows that the literature included in this survey is mainly dominated by optimization-centered workflows, especially evolutionary ones, and purely forward surrogate studies. In particular, the strong presence of surrogate-assisted evolutionary optimization, along with surrogate-assisted optimization, indicates that workflows based on optimization using surrogate models have formed the dominant functional pattern across the included studies. Furthermore, the notable presence of forward EM surrogate models confirms that a considerable portion of the literature still employs surrogate models, mainly for forward prediction, modeling, or analysis rather than for integrated design workflows.

The presence of active-learning EM frameworks and multifidelity surrogate frameworks further indicates that more advanced workflow structures are clearly represented in the included studies, although they did not dominate the taxonomy. In contrast, the remaining inverse surrogate model category is comparatively small. This should not be interpreted as evidence that inverse-design elements are uncommon across the corpus; instead, according to the fixed priority order of the taxonomy, studies on inverse-design elements, such as active-learning EM frameworks, multifidelity surrogate frameworks, and optimization-centered categories, were often placed in higher priority categories rather than remaining in the inverse surrogate model class [13,95,114].

The single study assigned to unified integrated pipelines should also be interpreted only within the taxonomy framework. In this review, the unified integrated pipelines were a formal taxonomy category assigned according to the decision hierarchy described in Section 2.4.5, and it was not equivalent to the unified-workflow condition examined in RQ 5. As discussed in Section 3.5, no fully classifiable study has simultaneously combined surrogate modeling, inverse design, multifidelity interaction, and active learning within the same workflow. Therefore, the presence of one study in the category of unified integrated pipelines does not contradict the earlier finding that no fully unified workflow was identified under the strict four-feature definition used for the unified-workflow question.

In summary, the taxonomy assignment complements the included studies. Although the individual workflow features and their combinations were examined in the previous subsections, the taxonomy indicates the most common dominant workflow structures when assigning each study to a single final category. Under this mutually exclusive classification, the included studies were dominated by optimization-centered and forward-surrogate workflow categories, whereas more strongly integrated taxonomy categories were less frequent.

### 3.7. Reporting Quality and Classification Transparency

This subsection briefly summarizes the reporting quality observed across the included studies in relation to the classification process described in Section 2.4.6.

Overall, the RF/microwave design target has been described clearly enough to support eligibility screening and scope classification. In most cases, it was possible to identify the component type, application context, and whether the study fell within the RF/microwave design scope of this review.

Additionally, the reporting of surrogate models was clear enough in most studies. In particular, the main surrogate family and its overall role in the workflow were usually described well enough to support the mapping table classification. In most cases, it was possible to determine whether surrogate models were mainly employed for forward prediction, optimization support, inverse design, or another clearly defined workflow role.

In contrast, the methodological workflow was not always reported with the same level of clarity. The most common ambiguities included whether optimization was truly specification-driven, whether multifidelity interaction was actually implemented, and whether adaptive sampling should be classified as formal active learning. These were the workflow elements that most often required careful interpretation during the classification process.

However, the reported methods and results were generally sufficient to support the technical characterization, taxonomy assignment, and the broader classification process adopted in this review. When a workflow element was not described clearly enough, only the attributes directly supported by the study were assigned, and the most conservative valid category was selected according to the rule-based procedure described in Section 2.

## 4. Discussion

This section discusses the main findings of this review in relation to the RQs, the adopted classification framework, and the broader context of surrogate-assisted RF/microwave design. Initially, we examine the main findings across the included studies. Then, we discuss the limitations of this review and the implications for future research.

### 4.1. Interpretation of the Main Findings

The results of this review indicate that surrogate-assisted RF/microwave design has been discussed in a wide variety of studies, but not consistently across the literature. Antennas and filters have dominated the relevant literature, whereas other component categories appeared less frequently. This indicates that surrogate-assisted RF/microwave design has been more strongly employed in applications where EM simulations are computationally intensive and iterative design is required.

When the included studies are examined from a workflow perspective, the clearest overall pattern is the strong use of surrogate models in optimization-centered design processes. This is reflected in the workflow feature-combination analysis (Figure 7) and even more clearly in the final taxonomy (Figure 8 and Table 2), where optimization-related categories account for the largest share of the fully classifiable studies. Representative studies include [23,29,53,78], where surrogate models were used to support parameter search and design refinement rather than forward prediction only. This pattern is compatible with the scope of a previous review on EM-driven optimization [4]. Our review examines this issue from a different perspective; it classifies the included studies according to the workflow structure across multiple RF/microwave component classes rather than considering optimization alone.

Another important finding is that inverse design is clearly present across the included studies; however, it mostly appears in optimization-based inverse workflows rather than in explicit inverse surrogate models. In many studies, the design methodology starts from the specifications of target electrical parameters and then uses surrogate-supported search to identify suitable design parameters [29,50,69,80]. In contrast, explicit inverse surrogate models that directly predict the geometry or design variables based on the specifications are much less common [57,92,105]. Hybrid inverse workflows are also present [43,49,51], but they are less frequent than optimization-based inverse workflows. Overall, the inverse design in the included studies is usually implemented via surrogate-supported search under predefined specifications rather than via direct inverse prediction alone.

Active learning and multifidelity methods are clearly present in the included studies; however, the most common workflow structure across the fully classifiable corpus is not represented. Active-learning studies include [19,87,121,130], and multifidelity studies include [10,32,59,60], indicating that these workflow elements are not absent; instead, they have not been widely adopted compared with the large group of surrogate-assisted optimization studies that do not explicitly include them. This is also relevant in a previous review on multifidelity learning in electromagnetics [6]. Our review specifically shows that within the RF/microwave component design, these strategies are clearly represented, but still do not form the most common workflow structure across the included studies.

Furthermore, the workflow feature-combination analysis and the final taxonomy consider the included studies from two different but complementary perspectives. The workflow feature-combination analysis (Figure 7) shows the workflow features that appear together within the same study, whereas the taxonomy (Figure 8 and Table 2) assigns each study to a main workflow category according to the decision order defined in Section 2.4.5. Consequently, inverse design elements appear more often across the full set of the included studies than suggested by the size of the inverse surrogate model category alone. For example, some studies that clearly include inverse-design logic are placed in higher priority categories, such as active-learning EM frameworks, multifidelity surrogate frameworks, or optimization-based categories [58,87,95]. This explains why the role of inverse design across the included studies is broader than the size of the inverse surrogate model category alone.

Compared with previous reviews, our review adds a more structured workflow perspective across RF/microwave component classes. ML in antenna design [2], broad EM applications [3], optimization-focused EM workflows [4], antenna-related AI and ML approaches [5], and multifidelity learning [6] have already been examined in previous reviews. However, based on their stated scope, those reviews have not employed the workflow classification used here. Our review complements the earlier literature by showing not only the methods used but also the functional positioning of surrogate models in RF/microwave design workflows, the frequency of simultaneously using inverse design, active learning, and multifidelity, as well as the main workflow integration gaps.

Overall, the studies included in this review contain many partial combinations of advanced workflow elements, but not the full combination addressed in RQ 5. Surrogate modeling combined with inverse design has been reported in many studies; in contrast, combinations that include active learning or multifidelity interaction are less frequent. Some studies have combined three of these elements within the same workflow. However, no fully classifiable study has implemented a single RF/microwave design workflow that combines surrogate modeling, inverse design, multifidelity interaction, and active learning. The single study assigned to unified integrated pipelines in the taxonomy [56] should be interpreted only within the taxonomy decision hierarchy and not as evidence of a fully unified workflow under the strict definition used in this review. Overall, our findings indicate that surrogate-assisted design is widely represented across the included RF/microwave design studies; however, the most advanced workflow elements are still used separately or only partially together rather than within a fully integrated workflow.

### 4.2. Limitations and Future Directions

This review has several limitations that should be considered when interpreting the findings. The evidence base was derived exclusively from the Scopus database. Although this provides a consistent and transparent basis for screening, relevant studies indexed in other databases may not have been captured. In addition, the search was restricted to English-language open access journal articles. As a result, conference papers, non-open access studies, and publications in other languages were outside the scope of this review. Conference publications on emerging design methods can be important in RF/microwave engineering. Furthermore, the final included corpus is limited by the availability and retrievability of full-text evidence. As discussed in Section 2.4.1, Ref. [47] was retained in the broader review corpus because the available abstract provided sufficient evidence that the eligibility criteria were satisfied; however, it was not included in the detailed technical classification, final taxonomy assignment, and workflow feature-combination analysis because the full text could not be retrieved. Finally, despite the predefined decision rules and the conservative classification approach, the final interpretation still depends on how clearly the workflow has been described in each study. When a workflow element was not reported clearly enough, the most conservative category was selected.

The above limitations also define the main future research directions. The results do not indicate that the required methodological elements are missing from the included studies. However, surrogate modeling, inverse design, multifidelity strategies, and active learning were all identified in the classified corpus. However, these elements were usually implemented separately or combined partially. The workflow feature-combination analysis shown in Figure 7 shows that all four elements have not been fully combined within the same implemented RF/microwave design workflow in any classifiable study.

A possible explanation is that multifidelity workflows are more demanding regarding the implementation of a stable and practically useful way; they require at least two model levels that are clearly related, along with a correction or updating approach that improves design efficiency while maintaining confidence in the final design result. In RF/microwave design, this involves combining low- and high-resolution EM models, circuit and full-wave models, or other representations with different levels of fidelity. This can become difficult when the response is strongly nonlinear or highly sensitive to geometry [26,59], and Ref. [114] illustrates how multifidelity strategies have been incorporated into RF/microwave design workflows. In the context of this review, such strategies are interpreted as examples of a more structured workflow integration rather than as simple extensions of a standard single-surrogate setup. Active learning may add another layer of workflow complexity. It requires not only retraining the surrogate model but also an explicit rule for selecting new samples, repeated interaction with the EM solver, and iterative updates of both the dataset and the model. In computationally intensive RF/microwave design problems, especially when optimization is already part of the workflow, this can increase the difficulty of setting up, controlling, and validating the overall process [72,95], and Ref. [117] confirms that active-learning strategies are present in the included studies; however, Figure 6a shows that they are much less common than workflows without active learning.

This can also explain why workflows based on optimization are the most common pattern across the fully classifiable studies. In many cases, a surrogate-assisted optimization process is relatively easier to define and fits naturally within the existing RF/microwave design practice. In contrast, a workflow combining specification-driven inverse design, adaptive sample selection, and multifidelity interaction within a single process is more demanding in terms of methodology and practical implementation. Consequently, compared with fully integrated frameworks that combine all advanced elements at once, the included studies describe workflows that improve design efficiency in a more modular and controlled way.

Overall, the results of this review do not suggest that the individual methods required for advanced automation are absent; instead, they show that these methods are still being used mostly in separate or only partially connected forms. This explains why the absence of the combined four-part methodology is important; it points to a meaningful methodological gap, not because the individual elements are missing, but because they have not yet been brought together into one clearly demonstrated RF/microwave design workflow within the classified corpus of this review.

## 5. Conclusions

In this systematic review, we examined surrogate-assisted EM design and optimization in the RF/microwave field. Specifically, we emphasized the use of surrogate models in design workflows rather than only in the reported ML models. In the Scopus-derived literature corpus, antennas and filters were the most frequently represented application areas; Gaussian process or Kriging models, as well as neural networks, were the most commonly reported surrogate families. At the workflow level, surrogate models have been mainly used in optimization-oriented design processes; in contrast, inverse models, multifidelity strategies, and active learning have appeared less frequently.

An important contribution of this review is that it provides a workflow-based synthesis of using surrogate models in RF/microwave design studies. Although inverse design, multifidelity interaction, and active learning were all present in parts of the included literature, no fully classifiable study has reported a single implemented workflow that combines surrogate modeling, inverse design, multifidelity interaction, and active learning. The findings indicate that future work should move beyond isolated or partially integrated strategies toward a complete workflow integration, particularly by combining specification-driven inverse design, adaptive sample selection, and multifidelity within a single RF/microwave design workflow.

## Figures and Tables

**Table 1:** Comparison of representative review articles and positioning of our review.

Review	Main Focus	Why This Review Is Useful	Why It Does Not Replace Our Review
[1]	Review of DL approaches for inverse-scattering problems	It summarizes DL methods for inverse scattering, discusses combinations of neural networks with underlying physics, and highlights challenges and limitations in inverse EM reconstruction.	It focuses on inverse-scattering problems rather than surrogate-assisted RF and microwave component design and optimization workflows.
[2]	Review of ML in antenna design and optimization	It focuses on ML methods for antenna design, including regression models, antenna synthesis, and antenna analysis.	It focuses on antenna design and does not provide a broad workflow synthesis across RF and microwave component classes.
[3]	Broad review of ML applications in electromagnetics	It provides a broad overview of ML applications across electromagnetics, including antenna design, inverse scattering, radar, sensing, and fault detection.	It is a broad application-level review rather than a synthesis focused on surrogate-assisted RF and microwave design workflows.
[4]	Review of EM-based optimization algorithms, including direct and surrogate optimization methods	It provides strong methodological coverage of EM optimization strategies, including surrogate model and ANN-based optimization approaches; examples include transmission lines, filters, and antennas.	It focuses on algorithm categories rather than on the workflow-level synthesis of surrogate-assisted design practice across RF and microwave component studies.
[5]	Review of AI/ML in antenna design, optimization, and measurement	It reviews recent AI/ML approaches for antenna-related design and measurement; additionally, it discusses associated challenges, limitations, and future opportunities.	It focuses on antenna design and discusses the broad AI/ML scope rather than being centered on surrogate-assisted RF and microwave design workflows across component classes.
[6]	Review of multifidelity learning approaches for EM problems	It focuses on multifidelity surrogate modeling for EM forward and inverse problems, including low-fidelity data generation and physics-based learning approaches.	It mainly focuses on the multifidelity methodology rather than the broader workflow-level synthesis of surrogate-assisted RF and microwave component design across component classes.
Our review	A systematic review of surrogate-assisted EM-driven design and optimization across RF and microwave component classes	It provides a structured synthesis of the application of surrogate-assisted methods across different RF and microwave component categories.	It complements previous specialized reviews by examining surrogate-assisted RF and microwave design practice across component classes within a single review framework.

**Table 2:** Final functional workflow taxonomy of the fully classifiable studies, showing the mutually exclusive category assignment, category-level counts and percentages, and the corresponding study references under the fixed decision hierarchy described in Section 2.4.5.

Representative References	Functional Workflow Taxonomy	Number of Studies	Percentage (%)
[56]	Unified Integrated Pipelines	1	0.8%
[22,127]	Hybrid Optimization Frameworks	2	1.6%
[57,92,93,105,115]	Inverse Surrogate Models	5	4.0%
[8,10,11,12,18,24,26,27,32,34,42,55,59,60,114,122]	Multifidelity Surrogate Frameworks	16	12.8%
[7,9,19,20,30,58,72,74,87,95,96,104,106,113,117,119,121,128,130]	Active Learning EM Frameworks	19	15.2%
[13,15,16,31,37,40,41,43,44,49,51,52,65,66,68,76,85,94,107,110,118,129]	Surrogate-Assisted Optimization	22	17.6%
[14,17,33,35,36,38,39,48,54,62,64,67,73,77,86,88,89,90,91,98,101,102,103,125,132]	Forward EM Surrogate Models	25	20.0%
[21,23,25,28,29,45,46,50,53,61,63,69,70,71,75,78,79,80,81,82,83,84,97,99,100,108,109,111,112,116,120,123,124,126,131]	Surrogate-Assisted Evolutionary Optimization	35	28.0%
	Total	125	

## Data Availability

The mapping, technical, taxonomy, and gap tables, as well as the graphical summaries for the included studies, are available in Supplementary Materials (Supplementary File S1).

## Supplementary Materials

The following supporting information can be downloaded at: https://www.mdpi.com/article/10.3390/s26082504/s1, Supplementary File S1: Excel file containing the mapping, technical, taxonomy, and gap tables, as well as the graphical summary sheets for the included studies.
